# Supplementary material for: Epitranscriptomic subtyping, visualization, and denoising by global motif visualization
Source: Nat Commun. 2023 Sep 23;14:5944. doi: 10.1038/s41467-023-41653-4 (PMC10517956; doi:10.1038/s41467-023-41653-4)
Supplement: Supplementary file 1 — Supplementary Information [file 41467_2023_41653_MOESM1_ESM.pdf]

Supplementary Information for

Epitranscriptomic subtyping, visualization, and denoising by global motif visualization

Jianheng Liu[1,2, *], Tao Huang[3, *], Jing Yao[1,*], Tianxuan Zhao[1], Yusen Zhang[1], Rui Zhang[1]

1. MOE Key Laboratory of Gene Function and Regulation, Guangdong Province Key Laboratory of Pharmaceutical Functional Genes, State Key Laboratory of Biocontrol, School of Life Sciences, Sun Yat-Sen University, Guangzhou, 510275, PR China
2. Department of Pharmacology, Weill Cornell Medicine, Cornell University, New York, NY, 10065, USA
3. Department of Pathology and Pathophysiology, Shantou University Medical College, Shantou, 515041, PR China

*These authors contributed equally to this work.

email: zhangrui3@mail.sysu.edu.cn (R.Z.); liujh26@mail2.sysu.edu.cn (J.H.L.)

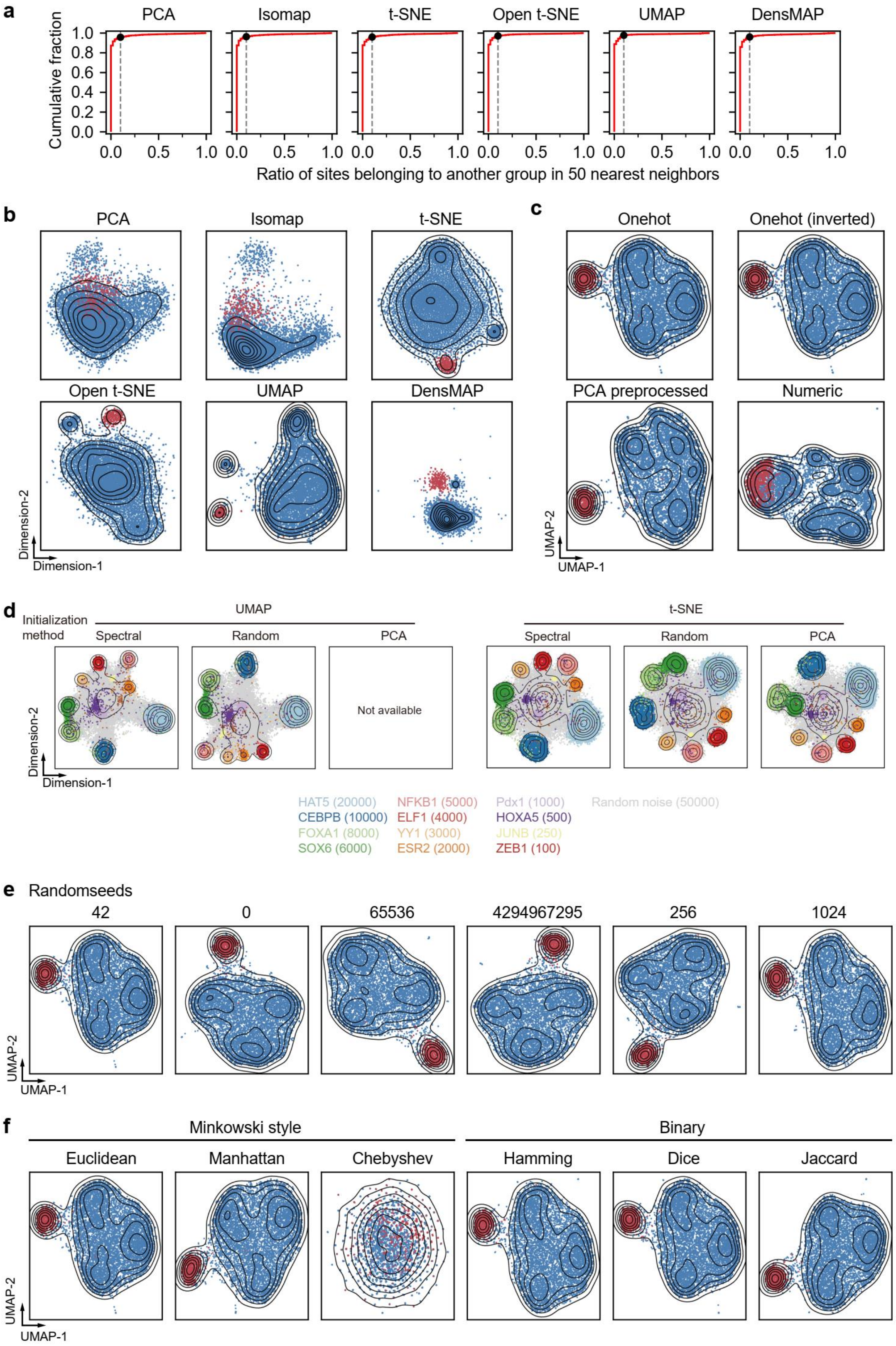

Supplementary Fig. 1. Further analysis of dimension reduction algorithms.

(**a**) The cumulative distribution of the ratios of sites belonging to another group in 50 nearest neighbors in different algorithms. Fly embryo data were used. The 90th

percentile values were shown in black dots.

(**b**) Comparison of dimension reduction algorithms using m^5C sites called from Noc-treated HeLa cells. Colors: blue, Type I sites; red, Type II sites. Note that Type II sites here could be further split into three groups (see later result section). The color codes are the same as in **b** throughout this figure.

(**c**) Testing UMAP with different sequence encoding methods. One-hot (inverted), the order of base type matrix of each base was inverted (e.g. [0, 0, 0, 1, 0] to [0, 1, 0, 0, 0]). PCA preprocessed, the one-hot encoded sequences were decomposed into 21 dimensions with PCA before sending to UMAP. Numeric, numeric labeling (e.g., A:0, U:1, C:2, G:3, N:4) was used to encode the sequence.

(**d**) The performance of UMAP and t-SNE with different initialization methods. The simulation dataset consists of 12 motifs. Two initialization methods in UMAP (spectral and random) and three initialization methods in Open-tSNE (spectral, random, and PCA) were tested. PCA initialization is unavailable for the current version of UMAP, although the parameter is shown in the manual. Different motifs were shown in colors. The numbers of motifs were shown in parentheses.

(**e-f**) Testing UMAP with different random seeds (**e**) or metric functions (**f**).

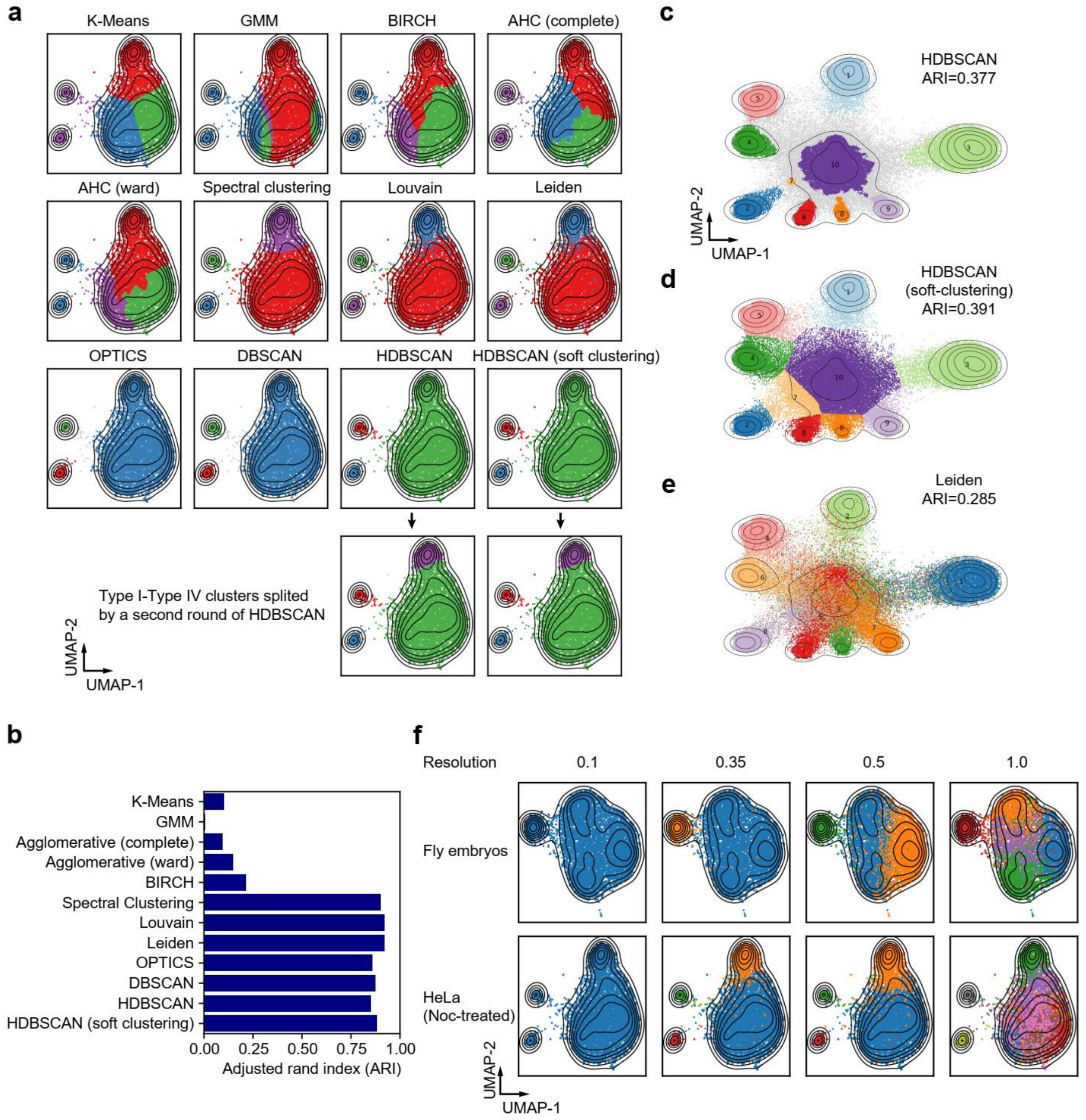

Supplementary Fig. 2. Further analysis of clustering algorithms.

(**a**) Comparison of clustering algorithms using m^{5}C sites called from Noc-treated HeLa cells. Clusters identified were labelled by different colors. Unclassified sites were shown in light gray. For HDBSCAN, both native clustering results and curated clustering results with a second round of HDBSCAN clustering on Type I-Type IV (see later result section for the definition of Type IV cluster) clusters were shown.

(**b**) The ARI scores in the fly embryo m^{5}C dataset.

(**c-e**) The clustering results and ARI scores of HDBSCAN, HDBSCAN (soft-clustering), and Leiden on the large simulation dataset. For Leiden, resolution parameter was set to 1 in this analysis.

(**f**) The clustering results with Leiden algorithm using m^{5}C sites called from fly embryos and Noc-treated HeLa cells. Different resolution parameters were used. Clusters identified were labelled by different colors. Similar results were obtained with Louvain algorithm (data not shown).

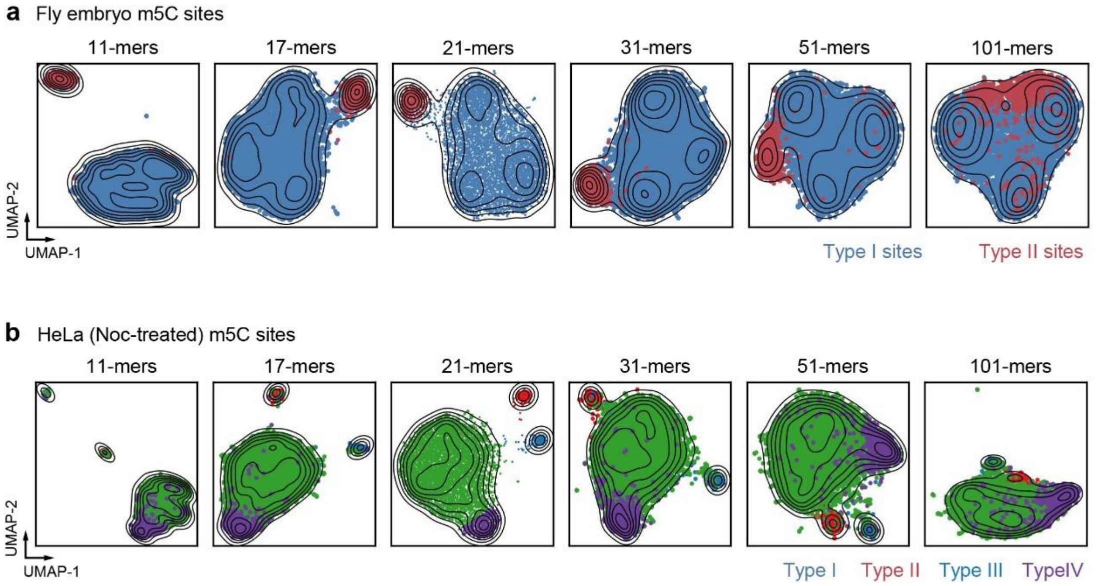

Supplementary Fig. 3. The impact of k-mer length selection on iMVP.

(**a-b**) iMVP was performed on 5, 8, 10, 15, 20, 25, and 50 nt flanking sequences (11 to 101 mers) of the m^{5}C sites found in fly embryos (**a**) or Noc-treated HeLa cells (**b**). Different clusters were shown in different colors.

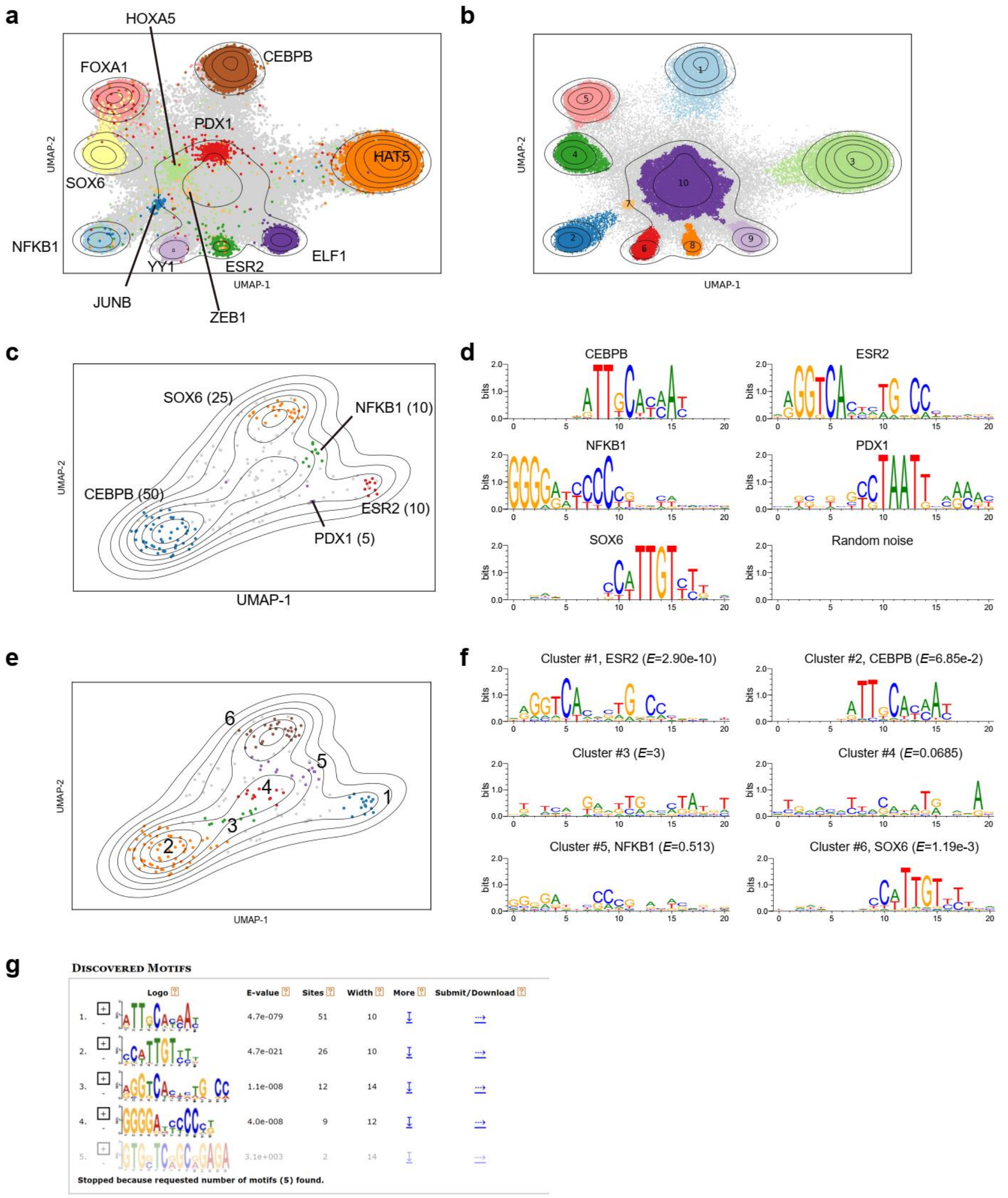

Supplementary Fig. 4. Comparison of iMVP with MEME.

(**a**) UMAP projection of the large simulation dataset (ground-truth). The simulated motifs were shown in different colors, random noises were shown in gray.

(**b**) iMVP analysis of the simulation data in (**a**).

(**c-d**) UMAP projection and motifs of the small simulation dataset.

(**e-f**) iMVP analysis and motifs for the small simulation dataset.

(**g**) MEME output for the small simulation dataset.

TF	Number	Ground truth	iMVP	MEME
HAT5	20000	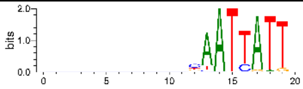	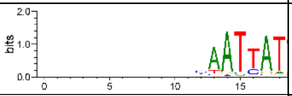	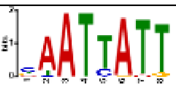
CEBPB	10000	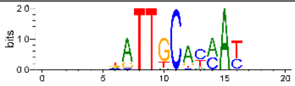	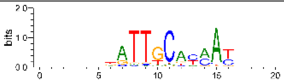	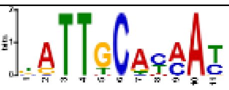
FOXA1	8000	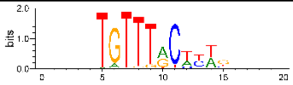	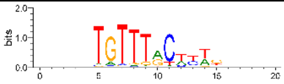	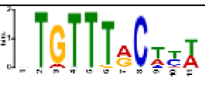
SOX6	6000	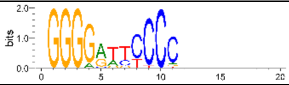	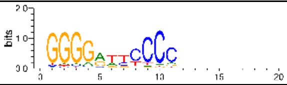	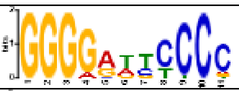
NFKB1	5000	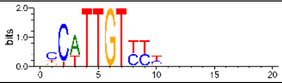	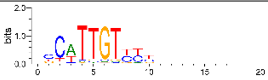	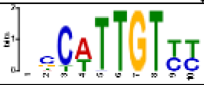
ELF1	4000	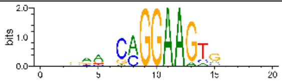	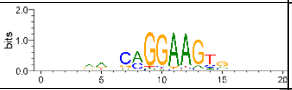	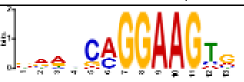
YY1	3000	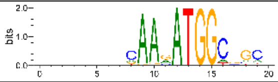	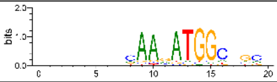	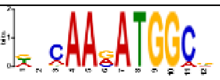
ESR2	2000	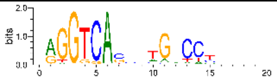	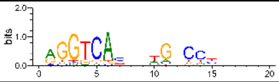	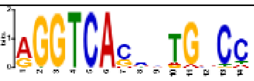
PDX1	1000	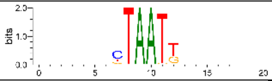	NA	NA
HOXA5	500	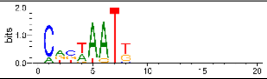	NA	NA
JUNB	250	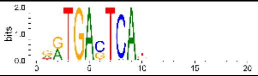	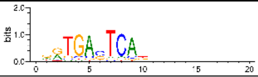	NA
ZEB1	100	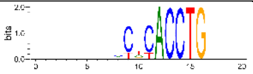	NA	NA
Cluster #10 (iMVP)			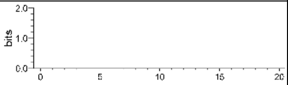	

Supplementary Fig. 5. Detailed information for the analyses on the large simulation dataset.

TF	Number	Ground truth	iMVP	MEME
CEBPB	50	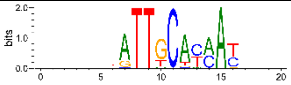	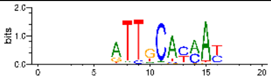	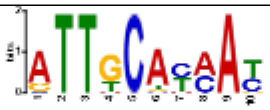
SOX6	25	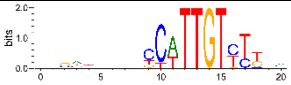	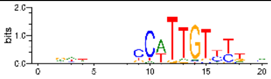	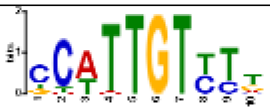
ESR2	10	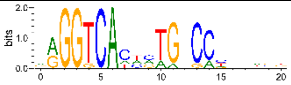	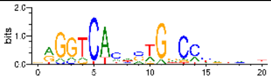	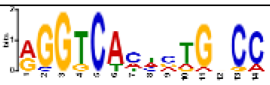
NFKB1	10	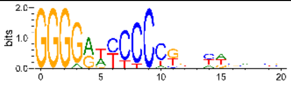	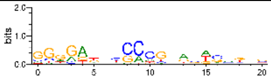	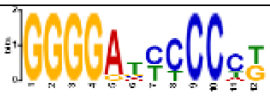
PDX1	5	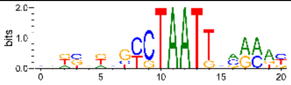		
Random noise	100	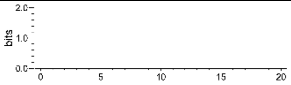		
iMVP cluster #3 (E= 1.14e-2)			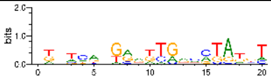	
iMVP cluster #4 (E=0.0685)			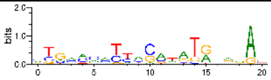	

Supplementary Fig. 6. Detailed information for the analyses on the small simulation dataset.

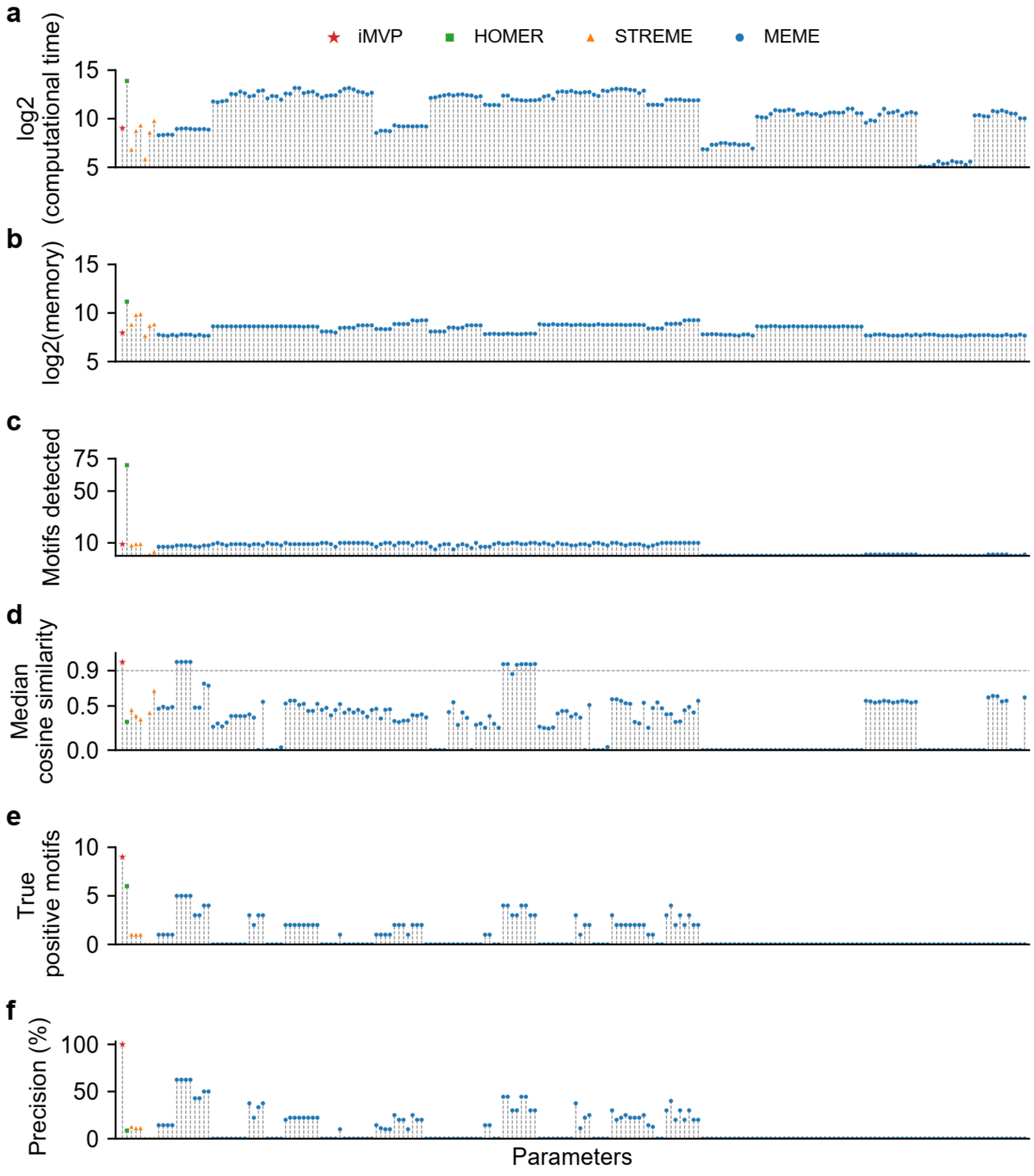

Supplementary Fig. 7. Comparison between iMVP and other motif search tools.

(**a**) Comparison of computational time among different methods. Red star, iMVP; green square, the combined result of 7 HOMER parameters; yellow triangle, six parameters of STREME; blue circle, 192 MEME parameters.

(**b**) Comparison of memory consumptions among different methods.

(**c**) Number of motifs detected by different methods.

(**d**) Median cosine similarity between motifs discovered by different tools and ground-truth motif position weights.

(**e**) Number of true-positive motifs (defined as the ones with cosine similarity greater than 0.9) found by different tools.

(**f**) Precision, defined as the number of true-positive motifs divided by the number of all motifs discovered, of different methods.

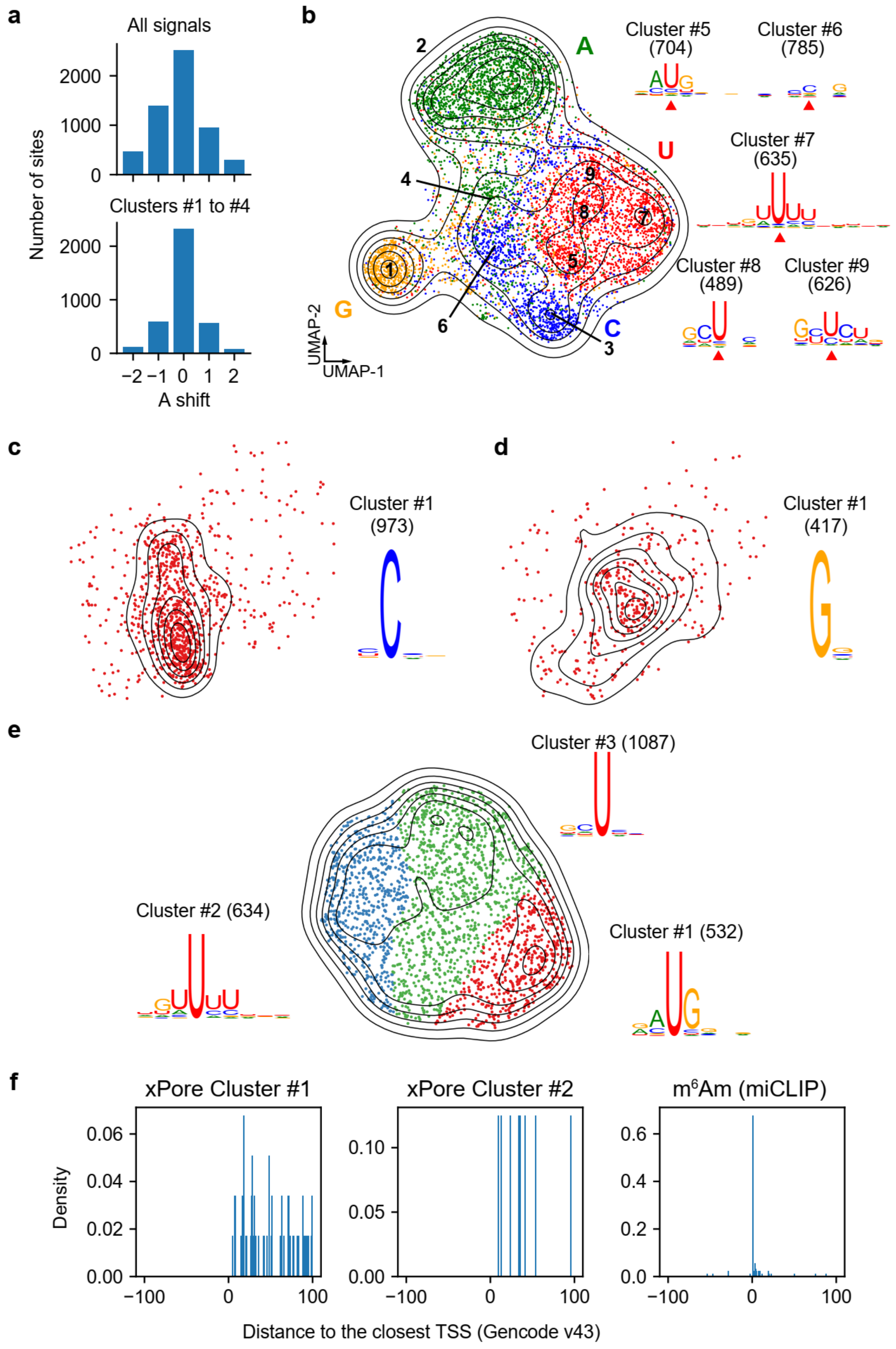

Supplementary Fig. 8. The subtypes of differential RNA modification sites identified by xPore.

(**a**) The number of A in different positions (phases) in sites identified by xPore. The

order of phase determination: 0 → -1 → +1 → -2 → +2.

(**b**) The locations of sites centered by A, U, C, and G bases in **Fig. 2b** and the corresponding motifs of clusters #5 to #9.

(**c-e**) iMVP analysis of sites centered by C, G, and U in **Fig. 2c.**

(**f**) Histograms showing the distributions of the distance between m^6A/m^6Am sites and the nearest TSSs. Cluster #1 was m^6A cluster and cluster #2 was CAR motif sites. m^6Am sites identified by miCLIP data (Linder et al. Nat Methods 12, 767–772 (2015)) were used as positive control.

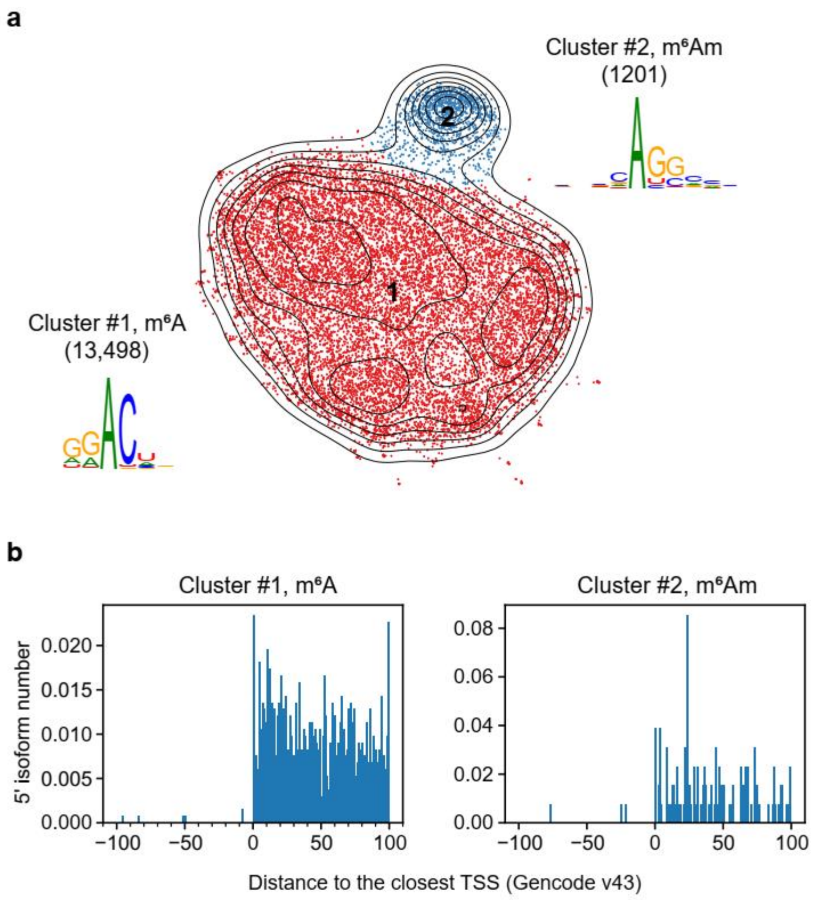

Supplementary Fig. 9. Analysis of m^{6}A/m^{6}Am sites called by m^{6}ACE-seq with iMVP.

(**a**) m^{6}A/m^{6}Am sites identified by m^{6}ACE-seq (Koh et al.) were used in this analysis. Cluster #1, putative m^{6}A sites within RRACH motifs; cluster #2, putative m^{6}Am sites within the CAR motif.

(**b**) Histograms showing the distributions of the distance between cluster #1/2 sites and the nearest TSSs.

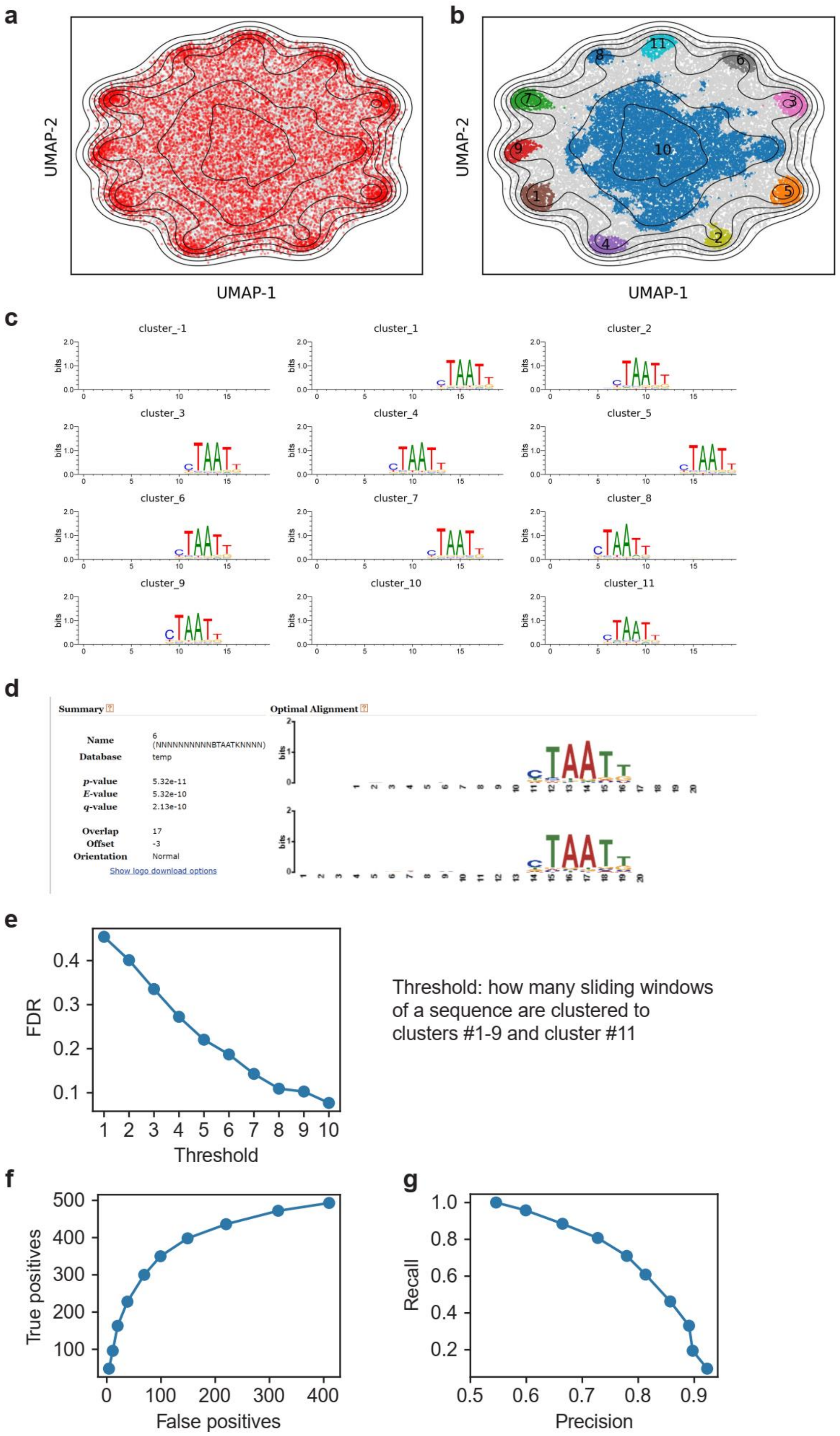

Supplementary Fig. 10. Sliding window strategy of iMVP on a simulation

dataset.

(**a**) The ground-truth X-Y locations of the sliding windows generated from simulated PDX1 motif containing sequences. PDX1 containing sequences were enriched at the edge of the UMAP projections.

(**b**) iMVP result for 30,000 sliding windows generated on 500 50 bp sequences containing PDX1 motif as well as 500 50 bp random noises.

(**c**) Motifs discovered by iMVP in (**b**).

(**d**) An example of motif alignment between cluster #1 and cluster #6 by Tomtom.

(e-g) The metrics for the sliding window strategy of iMVP. A sequence was considered to contain a PDX1 motif if it satisfied the criterion of having a certain number of sliding windows belonging to clusters #1-9 and cluster #11, as determined by a predefined threshold. Then metrics were calculated based on true positives, true negatives, false positives, and false negatives defined by the threshold. Three different metrics were present: threshold vs false discovery rate (FDR) **(e)**, number of false positives vs true positives **(f)** and precision vs recall **(g)**.

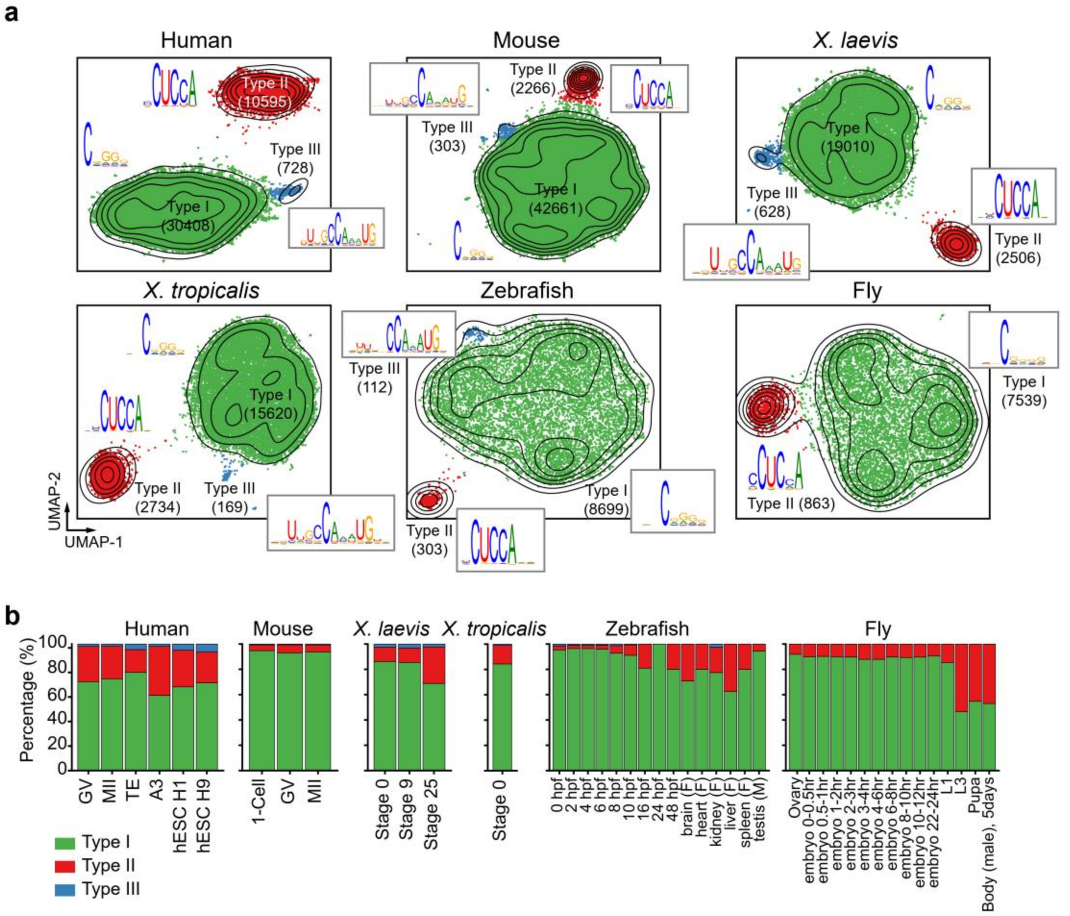

Supplementary Fig. 11. Analysis of the composition of m^{5}C sites in developmental stages.

(**a**) The global visualization of m^{5}C sites in early developmental stages of various species. The motifs of clusters agreed with the motifs of Type I, Type II, or Type III sites.

(**b**) The percentages of m^{5}C sites in each sample that belong to different m^{5}C types shown in (**a**).

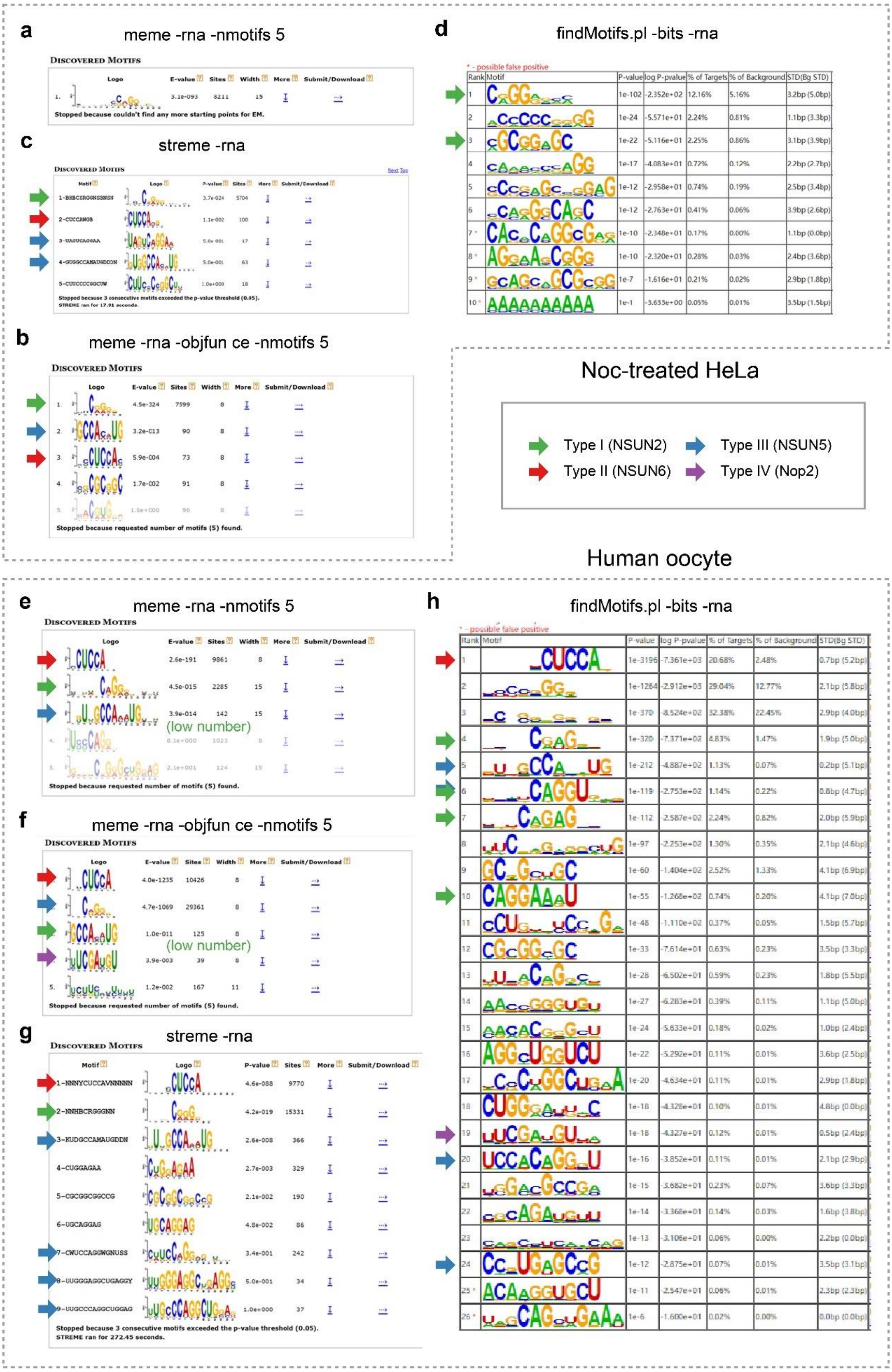

Supplementary Fig. 12. The Analysis of motifs of m^{5}C sites with MEME,

STREME, and HOMER.

(**a-d**) The motif search results of m^5C sites in Noc-treated HeLa cells.

(**e-h**) The motif search results of m^5C sites in human oocytes.

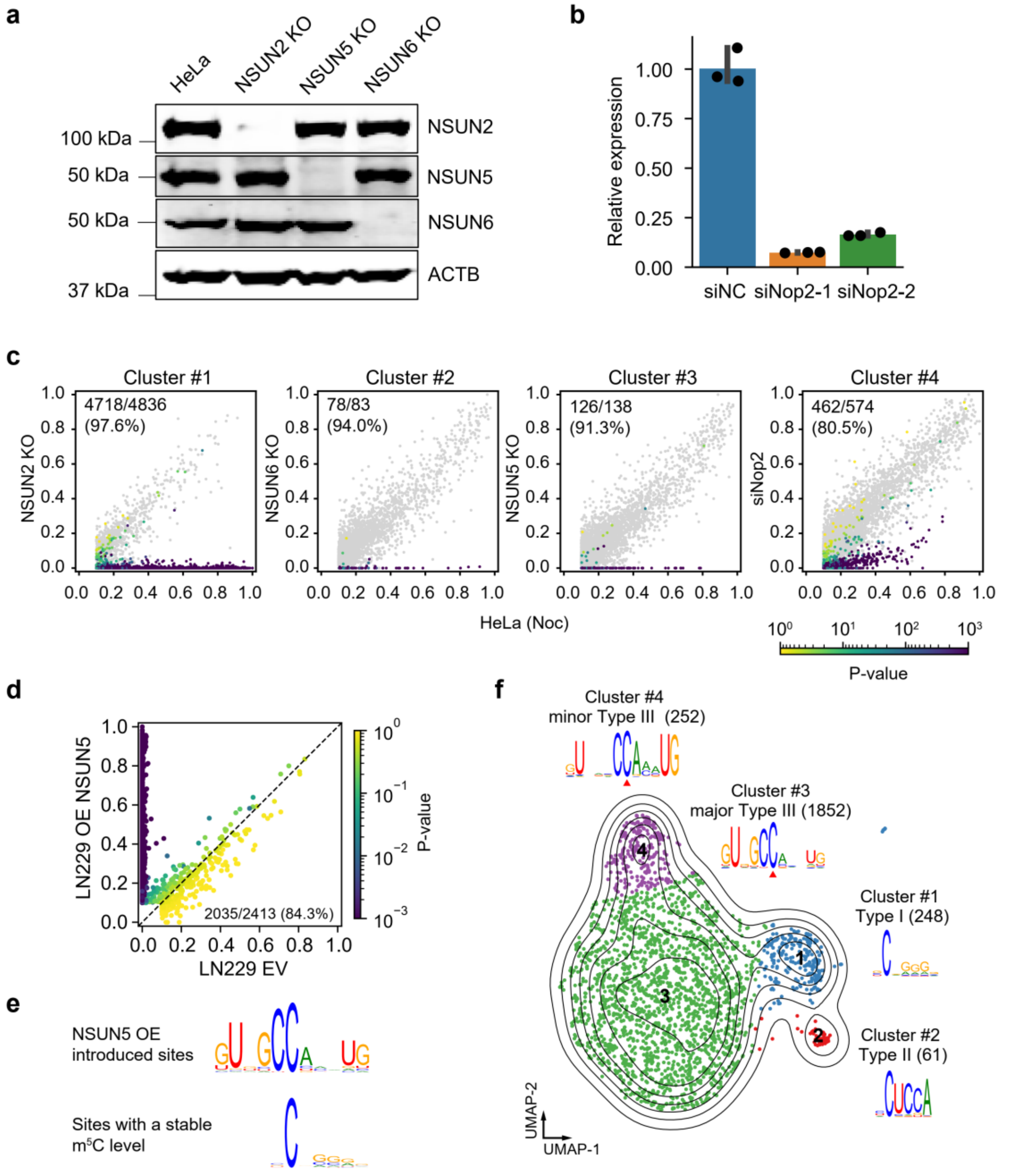

Supplementary Fig. 13. The validation of writers of Type III and Type IV sites.

(**a**) Western blotting validation of NSUN2, NSUN5, and NSUN6 knockout in HeLa cells. NSUN2 and NSUN6 knockout cells were obtained from our previous studies.

(**b**) qPCR validation of Nop2 knockdown via siRNAs in HeLa cells. Control gene, GADPH. Shown are sample means +/- S.D. n=3 biologically independent experiments.

(**c**) Comparison of m^{5}C methylation levels between Noc-treated NSUN2 KO, NSUN6 KO, NSUN5 KO, Nop2 KD HeLa cells and wild types cells for the corresponding

clusters. The number and percentage of sites with significantly decreased levels upon knockout or knockdown were shown. Sites covered by at least 20 reads in both samples and with methylation level ≥0.1 in wild type cells were analyzed. The P-values were calculated using one-sided Fisher's exact test.

(**d**) Comparison of m^5C methylation levels between NSUN5 overexpressed LN229 cells versus the cells transfected with empty vectors (EV). The number and percentage of sites with significantly increased levels upon NSUN5 overexpression were shown. Sites covered by at least 20 reads in both samples and with methylation level ≥0.1 in at least one sample were analyzed. The P-values were calculated using one-sided Fisher's exact test.

(**e**) The sequence context of C sites with (top) or without (bottom) significantly increased m^5C levels after NSUN5 overexpression.

(**f**) iMVP analysis of m^5C sites in (**d**). Two subtypes of Type III motifs were identified: cluster #3, the major Type III motif of 5'-GUNGCCANNUG-3'; cluster #4, the minor Type III motif of 5'-GUNNNCCAKHUG-3'. The methylated Cs of Type III motifs were indicated by red arrowheads. Type I and Type II motifs were also shown for cluster #1 and cluster #2.

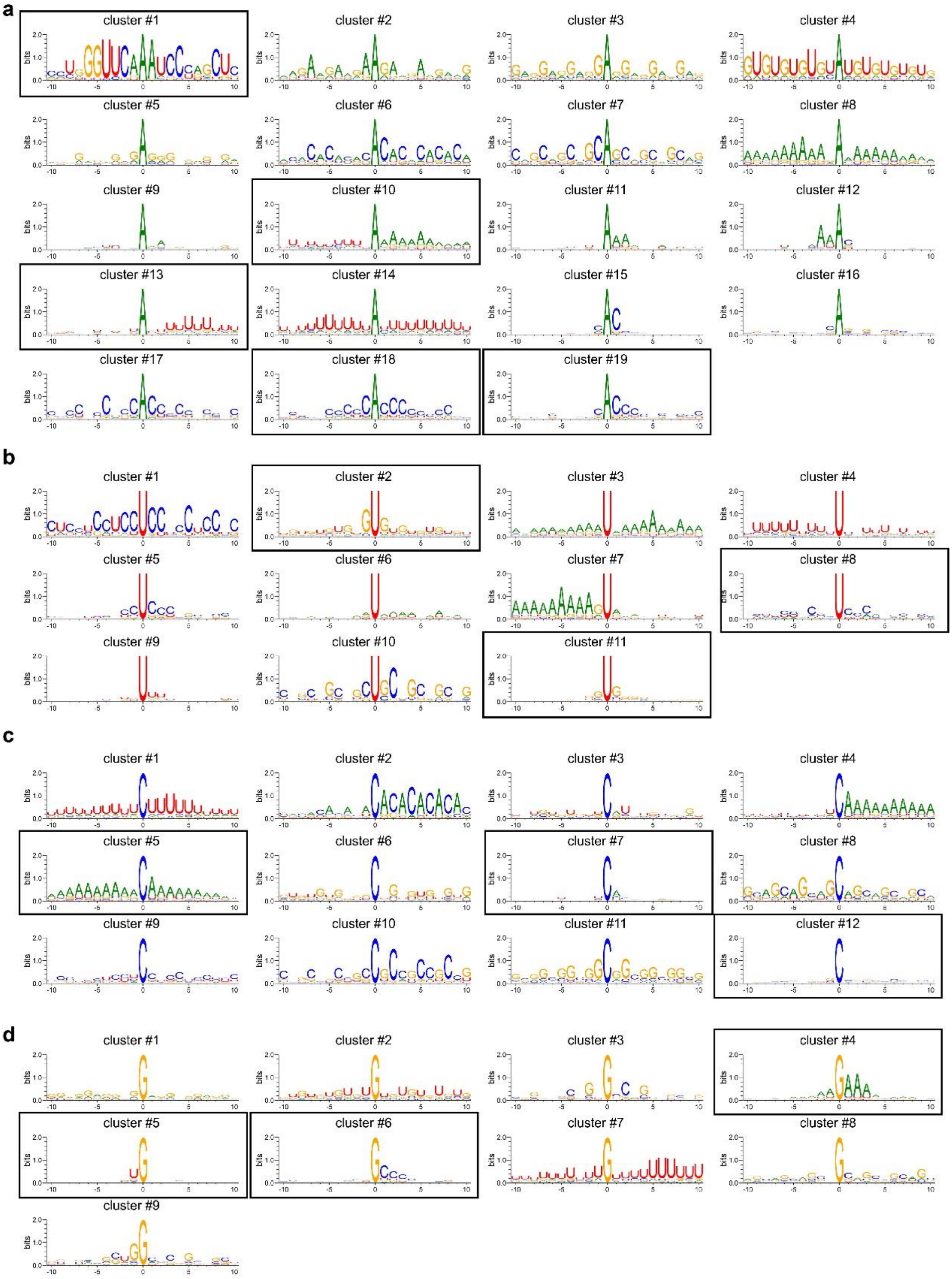

Supplementary Fig. 14. Motifs of ModTect clusters.

(**a-d**) Motifs of ModTect variants with A, U, C, and G as the reference base. Motifs shown in **Fig. 5** were highlighted with boxes.

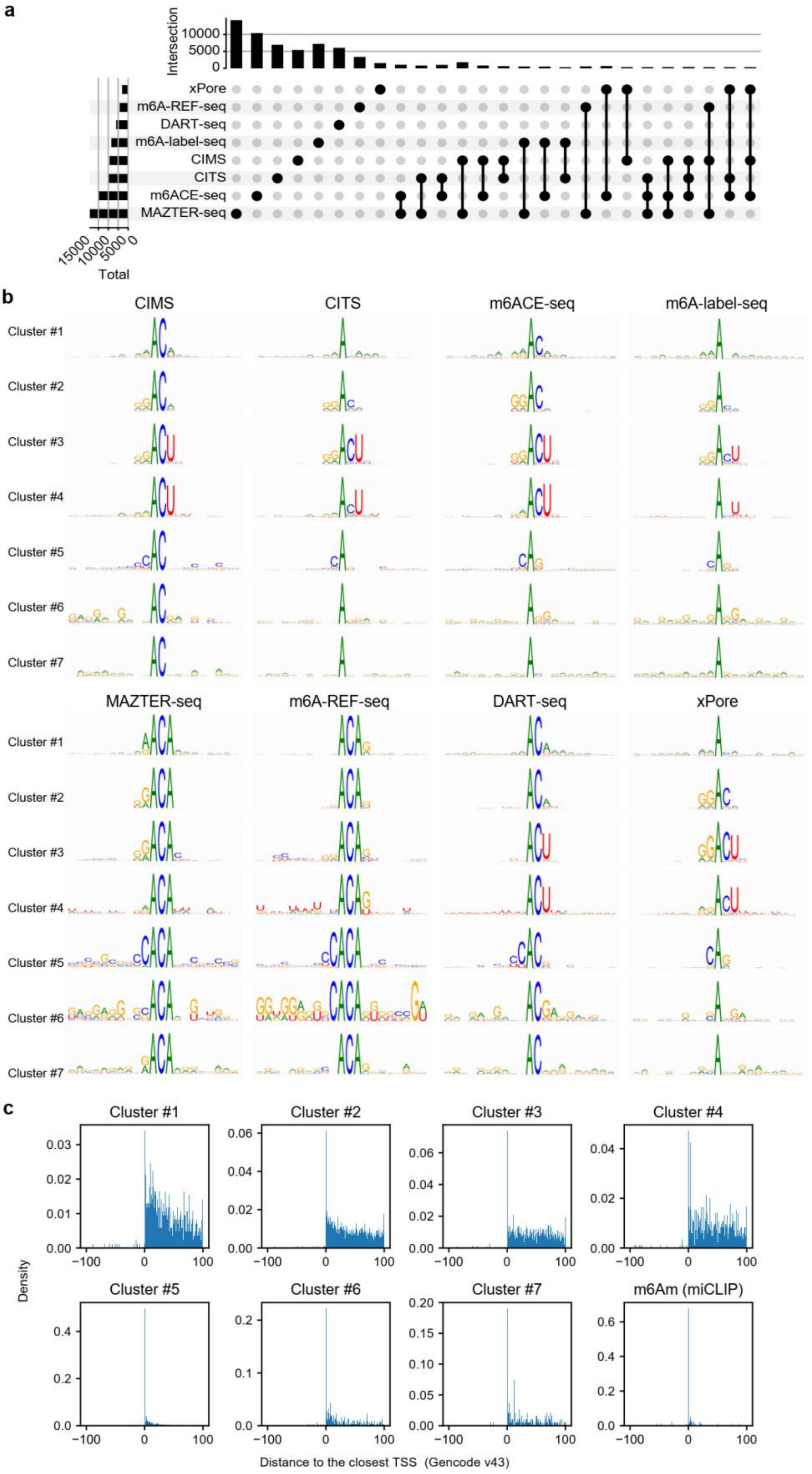
a
Intersection
10000
5000
0
xPore
m6A-REF-seq
DART-seq
m6A-label-seq
CIMS
CITS
m6ACE-seq
MAZTER-seq
15000
10000
5000
0
Total
b
CIMS
CITS
m6ACE-seq
m6A-label-seq
Cluster #1
Cluster #2
Cluster #3
Cluster #4
Cluster #5
Cluster #6
Cluster #7
MAZTER-seq
m6A-REF-seq
DART-seq
xPore
c
Cluster #1
Cluster #2
Cluster #3
Cluster #4
Cluster #5
Cluster #6
Cluster #7
m6Am (miCLIP)
Density
Distance to the closest TSS (Gencode v43)

Supplementary Fig. 15. Overlaps and motif comparison of sites between m^6A profiling methods.

(**a**) The UpSet plot showing the intersections among sites identified by different methods. Only intersections with at least 100 sites were shown.

(**b**) The motifs of each cluster of different methods.

(**c**) Histograms showing the distributions of the distance between m^6A/m^6Am sites and the nearest TSSs. Cluster #1 to #4 were m^6A clusters and cluster #5 was m^6Am-like cluster. m^6Am sites identified by miCLIP data were used as positive control. Cluster #1 to Cluster #4 could be served as negative control.

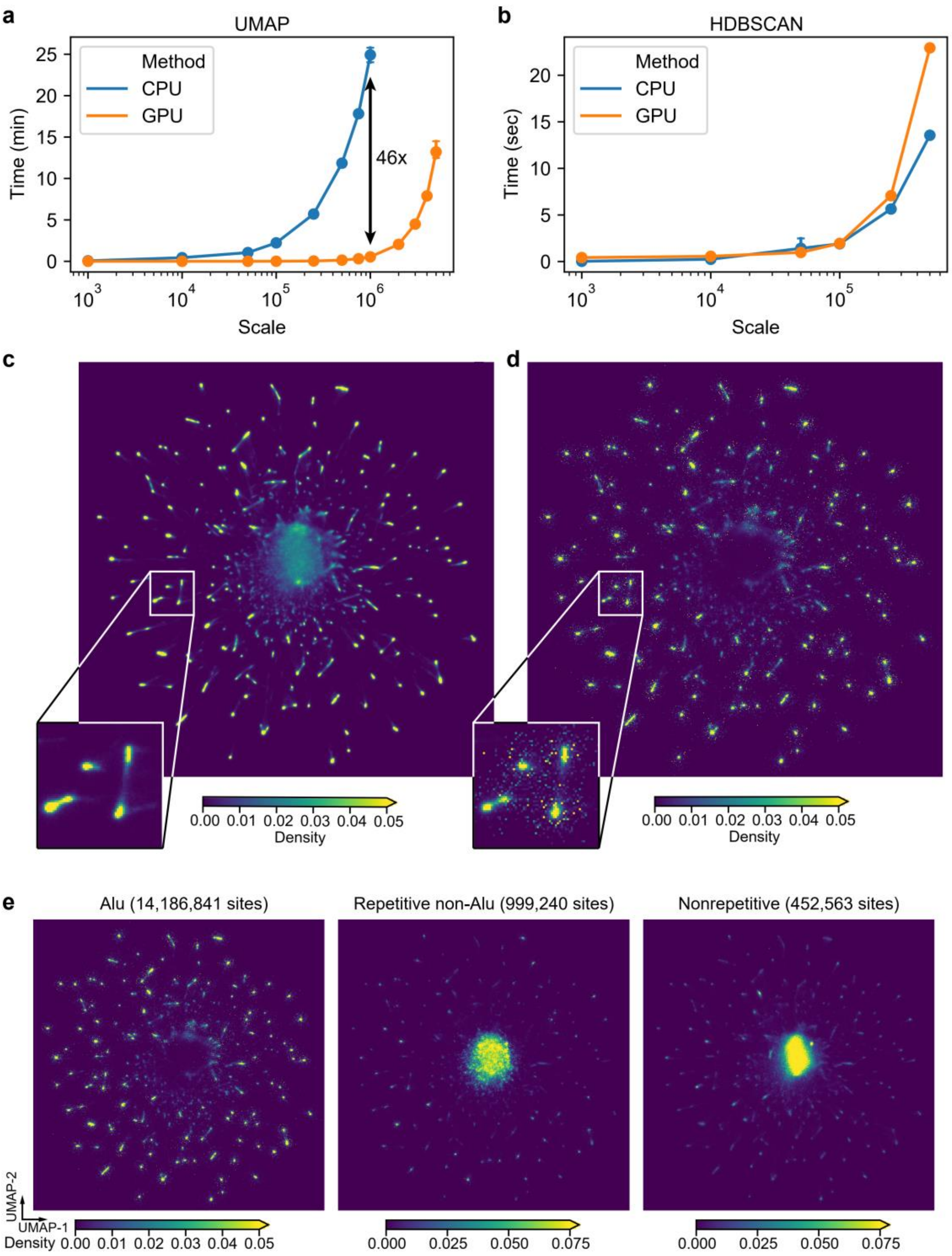

Supplementary Fig. 16. Benchmarking of GPU-accelerated iMVP.

(**a**) Comparison of the runtime between CPU- and GPU-based UMAP. Computation was repeated three times with fixed seeds to obtain the average runtimes and their 95% CI. For CPU-based UMAP, the impact of JIT was not included. UMAP

parameters: init=“random”, random_state=42, min_dist=0.01, n_neighbors=20; CPU-based only: n_jobs=6.

(**b**) Comparison of the runtime between CPU- and GPU-based HDBSCAN on the UMAP outputs obtained from (**a**). Computation was repeated three times to obtain the average runtimes and their 95% CI. HDBSCAN parameters: min_cluster_size=100, min_samples=100; CPU-based only, core_dist_n_jobs=6.

(**c**) The global visualization of all 5,081,915 different 21-mer sequences of human A-to-I RNA editing sites. A zoom-in view of some clusters was also shown.

(**d**) The global visualization of all 15,638,644 RNA editing sites. The coordinates of the projections of each site were retrieved from (**c**).

(**e**) The separate views of Alu, repetitive non-Alu, nonrepetitive sites in (**d**).

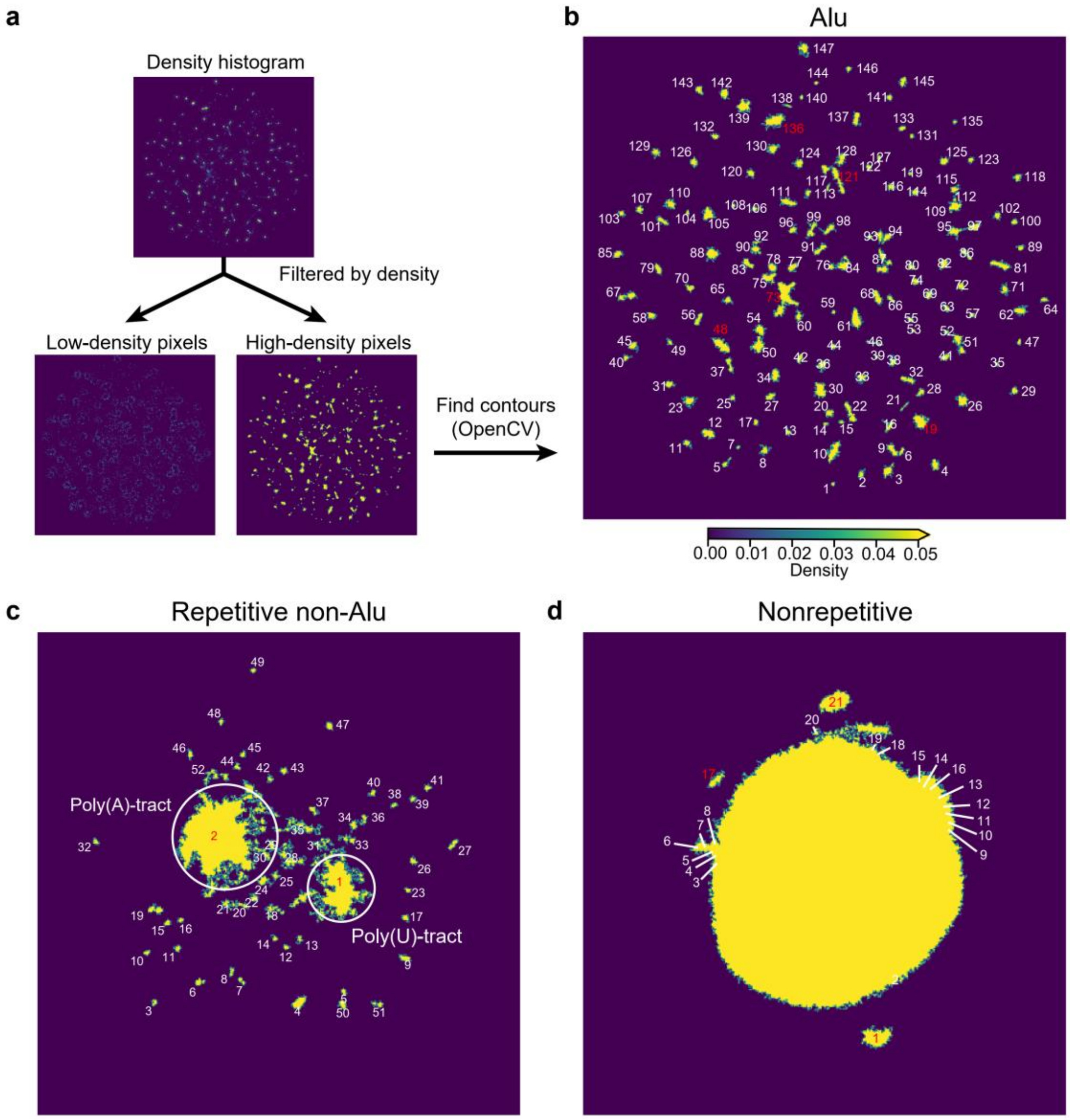

Supplementary Fig. 17. The Analysis of large clusters with density histogram based approximate clustering.

(**a**) The schema for approximate clustering. The density histogram was first filtered (de-noise) by the minimal density cutoff. Then the contours in the filtered histogram were extracted, and the sites were annotated by the clusters at pixel-level.

(**b**) The overview of clusters of Alu sites. Clusters shown in **Fig. 7c** were labeled in red.

(**c**) The overview of clusters in repetitive non-Alu sites. Cluster #1 and cluster #2 were poly(U)-tract and poly(A)-tract in **Fig. 7d**, respectively.

(**d**) The overview of clusters of nonrepetitive sites. Clusters shown in **Fig. 7e** were labeled in red.

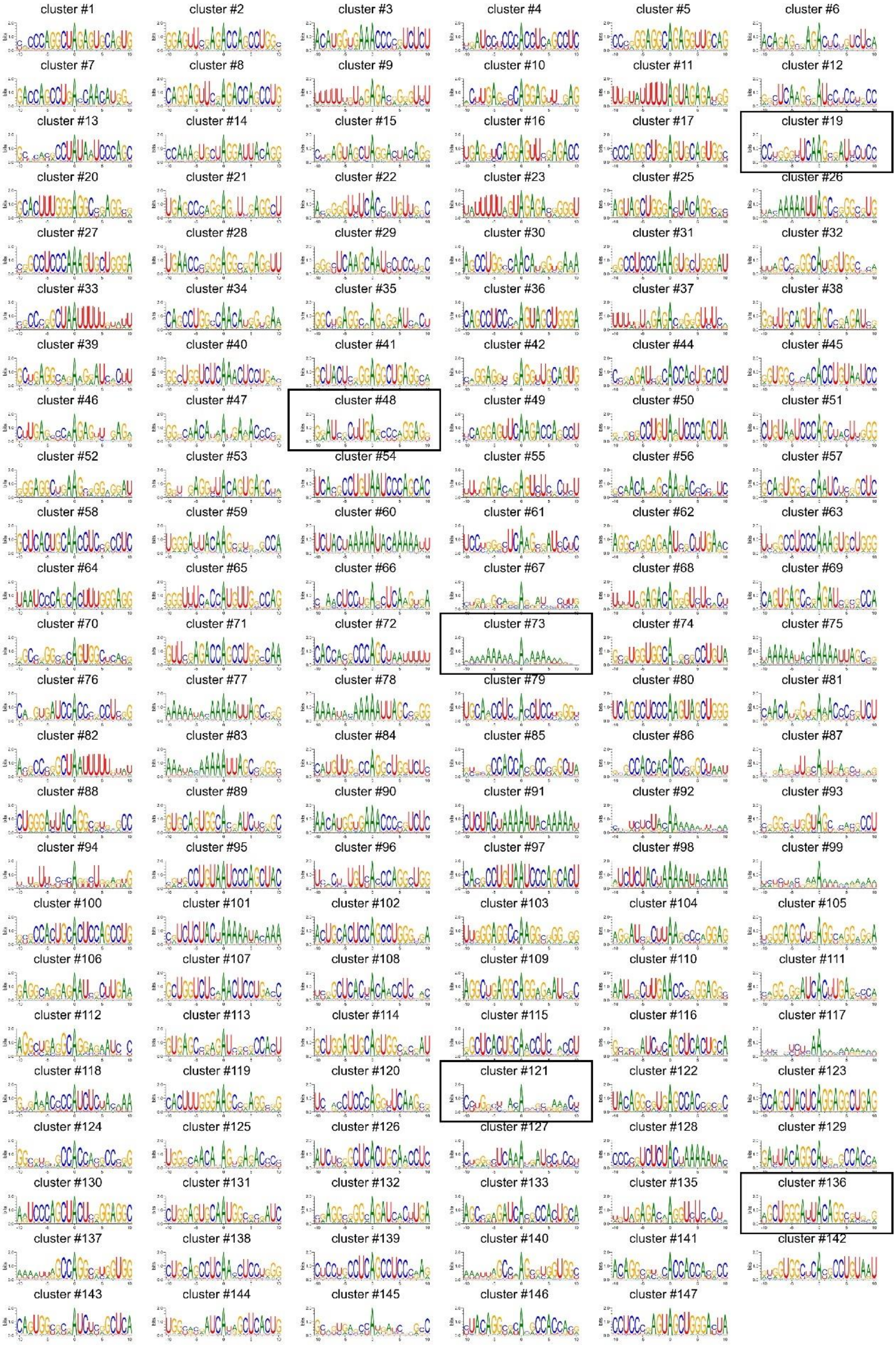

Supplementary Fig. 18. Motifs of clusters identified in Alu A-to-I editing sites.

Motifs shown in **Fig. 7** were highlighted with boxes.

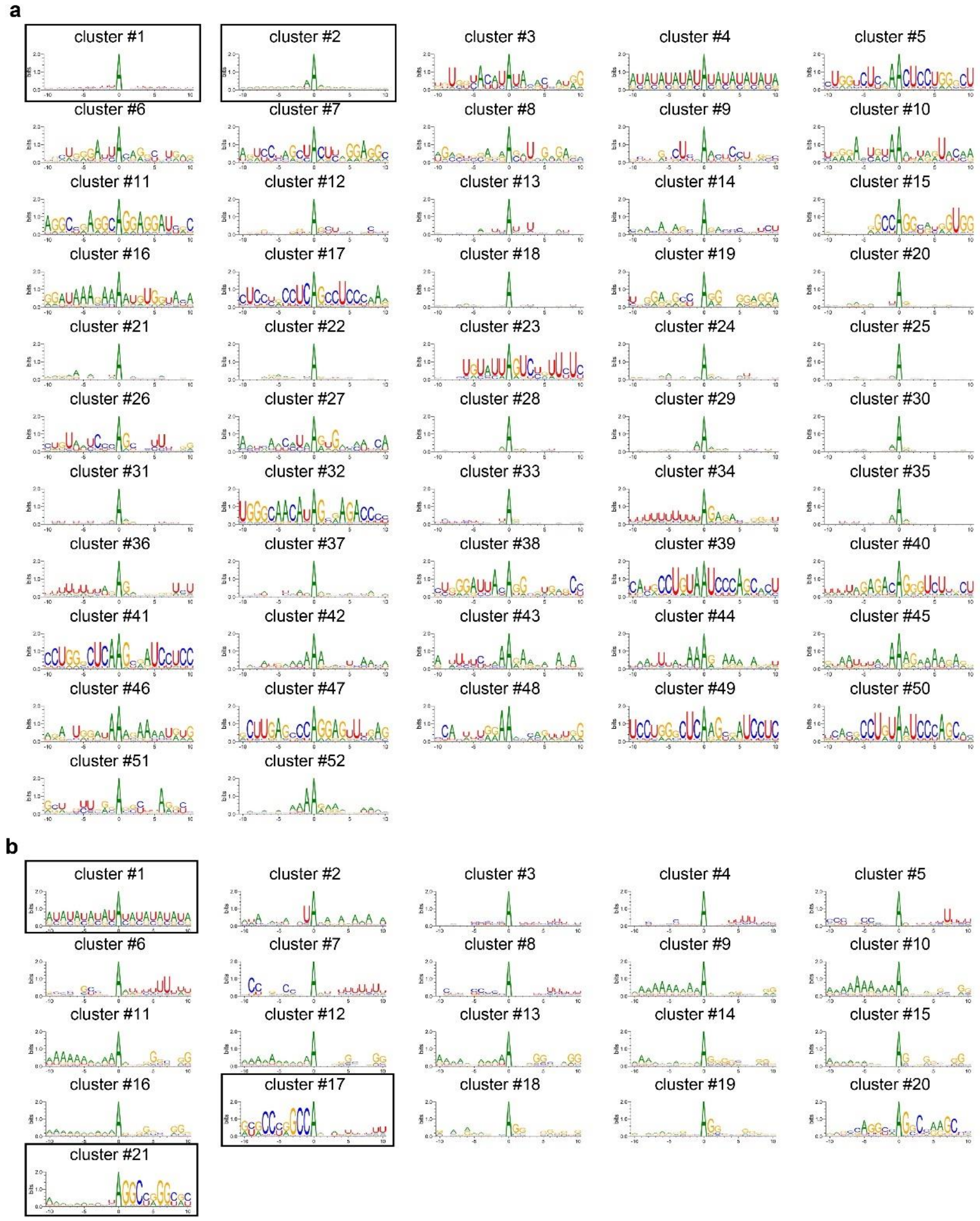

Supplementary Fig. 19. Motifs of clusters identified in repetitive non-Alu and nonrepetitive A-to-I editing sites.

(**a-b**) Motifs of repetitive non-Alu (**a**) and nonrepetitive (**b**) A-to-I editing sites. Motifs shown in **Fig. 7** were highlighted with boxes.

Supplementary Note 1. The generation of two simulation datasets.

The first dataset (marked as the large simulation dataset) contains 12 randomly chosen transcription factor motifs from JASPRA. The lengths of the motifs vary from 6 bp to 15 bp, and the numbers of motifs vary from 100 to 20,000. We also include 50,000 random noises. In total, this dataset contains 109,850 different sequences.

The second dataset (marked as the small simulation dataset) contains 5 motifs. The lengths of the motifs vary from 6 bp to 15 bp, and the numbers of motifs vary from 5 to 50. We also include 100 random noises. In total, this dataset has 200 different sequences.

Supplementary Note 2. The choice of algorithms.

UMAP and HDBSCAN outperformed other algorithms used for dimension reduction (e.g., PCA and DensMAP) and clustering (e.g., Louvain and Leiden) when analyzing datasets that were more complicated than the fly m^5C dataset, such as Noc-treated HeLa cells (**Supplementary Fig. 1-2**). Compared with UMAP, PCA failed to cluster the sites into 4 clusters and DensMAP was unable to set up a clear density contour between NSUN2-dependent and Nop2-dependent sites. For Louvain and Leiden, only one resolution parameter can be used in clustering. If this parameter was well-tuned (i.e., resolution=0.35), we might obtain good clusters. However, in most cases, we had either insufficient clustering (i.e., resolution=0.1) or over-clustering (i.e., resolution=0.5 and 1.0) results. Moreover, some sites were classified in interleaved clusters in Louvain and Leiden, which is inconsistent with the assumption that similar sequences should be close to each other on the 2-D plane.

Supplementary Note 3. The performance of iMVP and MEME in the large simulation dataset.

iMVP and MEME identified the enrichment of 9 and 8 of the 12 motifs, respectively. One of the least abundant motifs (JUNB) containing 250 records (1.8% of the dataset) can only be found by iMVP. Notably, with MEME, we had to specify the number of motifs to search for based on our prior knowledge (in this case, we set it to 12). However, with iMVP, no prior knowledge of motif number was required.
