# Supplementary material for: Epitranscriptomic subtyping, visualization, and denoising by global motif visualization
Source: Nat Commun. 2023 Sep 23;14:5944. doi: 10.1038/s41467-023-41653-4 (PMC10517956; doi:10.1038/s41467-023-41653-4)
Supplement: Supplementary file 3 — Description of Additional Supplementary Files [file 41467_2023_41653_MOESM3_ESM.pdf]

Description of Additional Supplementary Files

File Name: Supplementary Movie 1
Description: How to use an interactive version of iMVP.

File Name: Supplementary Data 1
Description: The list of Type I and Type II sites in fly early embryos.

File Name: Supplementary Data 2
Description: m^6A/m^6Am site list analyzed.

File Name: Supplementary Data 3
Description: m^5C site list in developmental stage samples of six species.

File Name: Supplementary Data 4
Description: m^5C sites in human cells and mouse tissues.

File Name: Supplementary Data 5
Description: ModTect variant lists.

File Name: Supplementary Data 6
Description: Summary of datasets used.

File Name: Supplementary Data 7
Description: Parameters of iMVP analysis used for different datasets.

File Name: Supplementary Data 8
Description: E-values, p-values, and q-values of the motifs presented in the study.
