# Supplementary material for: Epitranscriptomic subtyping, visualization, and denoising by global motif visualization
Source: Nat Commun. 2023 Sep 23;14:5944. doi: 10.1038/s41467-023-41653-4 (PMC10517956; doi:10.1038/s41467-023-41653-4)
Supplement: Supplementary file 13 — Reporting Summary [file 41467_2023_41653_MOESM13_ESM.pdf]

nature portfolio

Corresponding author(s): Rui Zhang

Last updated by author(s): Aug 20, 2023

Reporting Summary

Nature Portfolio wishes to improve the reproducibility of the work that we publish. This form provides structure for consistency and transparency in reporting. For further information on Nature Portfolio policies, see our Editorial Policies and the Editorial Policy Checklist.

Statistics

For all statistical analyses, confirm that the following items are present in the figure legend, table legend, main text, or Methods section.

n/a	Confirmed	
☐	☒	The exact sample size (*n*) for each experimental group/condition, given as a discrete number and unit of measurement
☐	☒	A statement on whether measurements were taken from distinct samples or whether the same sample was measured repeatedly
☐	☒	The statistical test(s) used AND whether they are one- or two-sided<br>*Only common tests should be described solely by name; describe more complex techniques in the Methods section.*
☒	☐	A description of all covariates tested
☐	☒	A description of any assumptions or corrections, such as tests of normality and adjustment for multiple comparisons
☐	☒	A full description of the statistical parameters including central tendency (e.g. means) or other basic estimates (e.g. regression coefficient) AND variation (e.g. standard deviation) or associated estimates of uncertainty (e.g. confidence intervals)
☐	☒	For null hypothesis testing, the test statistic (e.g. *F*, *t*, *r*) with confidence intervals, effect sizes, degrees of freedom and *P* value noted<br>*Give P values as exact values whenever suitable.*
☒	☐	For Bayesian analysis, information on the choice of priors and Markov chain Monte Carlo settings
☒	☐	For hierarchical and complex designs, identification of the appropriate level for tests and full reporting of outcomes
☒	☐	Estimates of effect sizes (e.g. Cohen's *d*, Pearson's *r*), indicating how they were calculated

Our web collection on statistics for biologists contains articles on many of the points above.

Software and code

Policy information about availability of computer code

Data collection	Illumina sequencing: Illumina HiSeq X Ten System 2 × 150 bp mode.
Data analysis	We set up iMVP analysis (CPU-based) with Python 3.7, and the following core packages were used: Pandas (v1.3.4), Numpy (v1.20.0), Scipy (v1.5.1), Scikit-learn (v0.23.1), biopython (v1.77), hdbscan (v0.8.27), umap-learn (v0.5.2), openTSEN (v0.6.1), louvain (v0.7.1), leidenalg (v0.8.8), dash (v2.2.0), dahs-bio (v0.9.0), imageio (v2.13.5), weblogo (v3.7.0), and opencv-python (v4.5.5). For GPU-based analysis, we used the Docker container provided by NVIDIA: RAPIDS release 22.02 in Ubuntu 18.04, Python 3.8, and CUDA 11.5. Additional analyses were performed with MEME (v5.0.0), MotifSuite, Weblogo (v3.7.0), MEME/ STREME (v5.3.3), HOMER (v4.11), RNAfold (v2.4.12), Cutadapt (v1.8.1), Trimmomatic (v0.36), HISAT2 (v2.2.0), Bowtie2 (v2.5.0). The source code is published at https://github.com/SYSU-zhanglab/iMVP.

For manuscripts utilizing custom algorithms or software that are central to the research but not yet described in published literature, software must be made available to editors and reviewers. We strongly encourage code deposition in a community repository (e.g. GitHub). See the Nature Portfolio guidelines for submitting code & software for further information.

Data

Policy information about availability of data

All manuscripts must include a data availability statement. This statement should provide the following information, where applicable:

- Accession codes, unique identifiers, or web links for publicly available datasets
- A description of any restrictions on data availability
- For clinical datasets or third party data, please ensure that the statement adheres to our policy

All sequencing data are available in the GEO database under accession GEO: BS-seq of HeLa cells with NSUN protein deficiency: GSE197650; control HeLa cells: GSM5319029; m6A-seq data generated in this study: GSE198955.
The source code is published at https://github.com/SYSU-zhanglab/iMVP.

Human research participants

Policy information about studies involving human research participants and Sex and Gender in Research.

Reporting on sex and gender	n/a
Population characteristics	n/a
Recruitment	n/a
Ethics oversight	n/a

Note that full information on the approval of the study protocol must also be provided in the manuscript.

Field-specific reporting

Please select the one below that is the best fit for your research. If you are not sure, read the appropriate sections before making your selection.

☒ Life sciences ☐ Behavioural & social sciences ☐ Ecological, evolutionary & environmental sciences

For a reference copy of the document with all sections, see nature.com/documents/nr-reporting-summary-flat.pdf

Life sciences study design

All studies must disclose on these points even when the disclosure is negative.

Sample size	Sample size was chosen based on material available ensuring that it will be appropriate for statistical analysis.
Data exclusions	no data exclusion.
Replication	The mean and 95% CI were calculated based on three replications.
Randomization	Randomization was used during the generation of simulation datasets: 1. We utilized a simulation dataset comprising 500 PDX1 motifs randomly distributed in a 50-bp sequence, along with 500 random noise sequences. 2. The large simulation dataset contains 12 randomly chosen transcription factor motifs from JASPRA. We also include 50,000 random noises. 3. The small simulation dataset contains 5 motifs. We also include 100 random noises.
Blinding	No blinding process was required, being a comupational tool.

Reporting for specific materials, systems and methods

We require information from authors about some types of materials, experimental systems and methods used in many studies. Here, indicate whether each material, system or method listed is relevant to your study. If you are not sure if a list item applies to your research, read the appropriate section before selecting a response.

Materials & experimental systems

n/a	Involved in the study
☐	☒ Antibodies
☐	☒ Eukaryotic cell lines
☒	☐ Palaeontology and archaeology
☒	☐ Animals and other organisms
☒	☐ Clinical data
☒	☐ Dual use research of concern

Methods

n/a	Involved in the study
☒	☐ ChIP-seq
☒	☐ Flow cytometry
☒	☐ MRI-based neuroimaging

Antibodies

Antibodies used

The primary antibodies used are anti-NSUN2 polyclonal antibody (Proteintech, 20854-1-AP, lot: 00091779, 1:1000 dilution in 5% milk-TBST), anti-NSUN6 polyclonal antibody (Proteintech, 17240-1-AP, lot: 00095057, 1:1000 dilution in 5% milk-TBST), anti-NSUN5 polyclonal antibody (Proteintech, 15449-1-AP, lot: 00059605, 1:1000 dilution in 5% milk-TBST), anti-m6A polyclonal antibody (SYSY, 202003, lot: 2-87, 1:50 dilution in IPP buffer) and anti-ACTB monoclonal antibody (ZSGB-BIO, TA-09, lot: 201050923, 1:2000 dilution in 5% milk-TBST). The secondary antibodies used are IRDye 800CW Goat-anti Mouse polyclonal antibody (LI-COR, 926-32210, lot:D00311-03,1:10000 dilution in 5% milk-TBST) and IRDye 680RD Donkey anti-Rabbit polyclonal antibody (LI-COR, 926-68023, lot: D10209-15, 1:10000 dilution in 5% milk-TBST).

Validation

The NSUN2, NUSN5 and NSUN6 antibodies were validated by knockout cell lines. The m6A antibody have been validated by the vendors.

Eukaryotic cell lines

Policy information about cell lines and Sex and Gender in Research

Cell line source(s)

HeLa and HEK293T cells were purchased from Cell Bank, Type Culture Collection, Chinese Academy of Sciences (CBTCCCAS).

Authentication

These cell lines have been identity verified using STR analysis by CBTCCCAS.

Mycoplasma contamination

These cell lines are routinely tested for mycoplasma by PCR detection of conditioned medium and all are tested negative.

Commonly misidentified lines
(See ICLAC register)

No commonly misidentified cell lines used.
